# The role of identity priming on the (unconscious) bodily self-attribution

**DOI:** 10.1007/s00426-024-01944-x

**Published:** 2024-03-16

**Authors:** Tommaso Ciorli, Lorenzo Pia

**Affiliations:** 1 SAMBA (SpAtial, Motor and Bodily Awareness) Research Group, Department of Psychology, University of Turin, Via Verdi 10, 10123 Turin, Italy; 2 NIT (Neuroscience Institute of Turin), Turin, Italy

## Abstract

It has been recently demonstrated that hand stimuli presented in a first-, with respect to a third-, person perspective were prioritized before awareness independently from their identity (i.e., self, or other). This pattern would represent an unconscious advantage for self-related bodily stimuli rooted in spatial perspective. To deeper investigate the role of identity, we employed a breaking-Continuous Flash Suppression paradigm in which a self- or other-hand presented in first- or third-person perspective was displayed after a conscious identity-related prime (i.e., self or other face). We replicated the unconscious advantage of the first-person perspective but, crucially, we reported that within the first-person perspective, other-hand stimuli preceded by other-face priming slowed down the conscious access with respect to the other conditions. These findings demonstrate that a top-down conscious identity context modulates the unconscious self-attribution of bodily stimuli. Within a predictive processing framework, we suggest that, by adding ambiguous information, the prime forces a prediction update that slows conscious access.

## Introduction

Visual processing of corporeal stimuli allows to quickly create judgements related to posture, age, ethnicity, gender but also ownership (i.e., attributing the seen body/body parts to the self or to another person). Ownership attribution for visually-presented corporeal stimuli is indeed a key biological signature of human species being necessary to actively move in the surrounding space and to interact with the others (Kaiser et al., 2008; Kessler & Thomson, 2010).

It is well-known that spatial perspective and identity are two fundamental primitive cues subserving ownership attribution of bodily stimuli (Chan et al., 2004; De Bellis et al., 2017). The former refers to the vertical orientation of the stimulus (i.e., upside down or upright), while the latter is related to its visual appearance. With respect to perspective, it known that hand images, for instance, presented in a first-person (i.e., upright orientation with fingers up), are typically attributed to the own body, whereas those in a third-person (i.e., upside down orientation, with fingers downward) are typically attributed to someone else (Brady et al., 2011; Choisdealbha et al., 2011; Conson et al., 2010). Such behavioral dissociation is underpinned by different cortical activations (Carey et al., 2019; Chan et al., 2004; Saxe et al., 2006). As regards identity, it is known that the visual resemblance of a seen hand with one’s own actual hand, as compared to somebody else’s’ hand, increases the attribution of the stimulus to the own body (Pyasik et al., 2020; Ratcliffe & Newport, 2017). Consistently, some studies have reported that brain areas subserving the visual processing of hands belonging to the self differ from those activated when hands belong to another person (De Bellis et al., 2017; Hodzic et al., 2009a; Hodzic et al., 2009b; Myers & Sowden, 2008; Orfei et al., 2007; Pann et al., 2021). Taken together, all these findings suggest a possible functional dissociation between these two important cues. Moreover, since there is a large consensus that vision is the most relevant sense, it is fundamental to understand whether and how the two above-mentioned cues impact ownership attribution. A recent study (Ciorli & Pia, 2023) investigated whether and to which extent identity and perspective affected ownership attribution at early levels of visual processing. The authors capitalized on the breaking-Continuous Flash Suppression (bCFS) paradigm (Jiang et al., 2007; Stein et al., 2011) in which, by decreasing the contrast of a mask flashed to one eye that initially suppresses a target stimulus shown to the other, the target becomes visible. It is assumed that stimuli overcoming faster the mask suppression share an earlier processing outside awareness with respect to those that need longer time. In other words, the paradigm employs a direct index of conscious perception (i.e., the detection time) to infer the timing for unconscious processing, more specifically the timing for conscious access. By comparing self- and other-hands visual stimuli presented in first- or third-person spatial perspective, the authors of the study (Ciorli & Pia, 2023) found that only spatial perspective affected visual awareness prioritization so that the first-person speeded up the access to consciousness. The authors argued in favor of an earlier unconscious prioritization of an egocentric body coding necessary for action monitoring. Crucially for the present study, they speculated that the absence of any identity (self vs. other) effect could be attributed to the fact that such feature relies on different or/and higher cognitive processes not indexed by that paradigm. However, besides the fact that an absence of evidence is not the evidence of absence *per se* (Altman & Bland, 1995), there is a body of literature that, although usually with different paradigms, gives a certain role to identity in visual processing of corporeal stimuli. For instance, it has been demonstrated that humans are more accurate to implicitly recognize the own hand with respect to somebody else’s hand (Frassinetti et al., 2011).

According to the above-mentioned considerations, here we aimed to investigate more deeply the role of identity on ownership attribution, specifically the self-relevance prioritization effect. To do so, we capitalized on the phenomenon of priming, that is the improvement in accuracy or speed responses to stimuli when they are preceded by a related stimulus that pre-activate associated information (Maljkovic & Nakayama, 1994). Specifically, we investigated the impact of top-down identity context on the conscious access of hand stimuli belonging to the participant or to another person, presented in a first- or third-person perspective. We capitalized on the bCFS paradigm, being a suitable paradigm useful to investigate the influence of contextual factors on the timing for visual awareness of invisible stimuli (Gayet et al., 2014; Ciorli et al., 2024). Indeed, it has been demonstrated a faster conscious access of stimuli when preceded by consciously perceived congruent primes at multiple levels, such as semantic (Costello et al., 2009), multimodal (Alsius & Munhall, 2013), memory (Gayet et al., 2013) - but see Stein and colleagues (Stein et al., 2023) - processing. In line with these considerations, we designed a bCFS experiment in which each trial was preceded by an identity-related prime (i.e., self/other-faces) to test whether activating the self/other representation in a top-down fashion would influence the access to awareness of hand stimuli with a given perspective (i.e., first/third) and identity (i.e., self/other). Congruently with the fact that top-down context plays a role in self-recognition, self-other distinction (Apps & Tsakiris, 2014), and conscious perception (Gilbert & Li, 2013), we hypothesized that face primes would have prioritized the breaking of congruent hand stimuli in visual awareness, whereas conscious access would have been slower in incongruent conditions.

### Participants

Twenty-six (eighteen females, mean age = 23 ± 3 years) right or left-handed (*n* = 2) participants (self-report) with normal or corrected-to-normal vision participated in the study. Sample size was decided according to a published study with the same paradigm and similar design (Weng et al., 2019). Nevertheless, to assess the minimum effect size our sample could detect, we computed a post-hoc sensitivity power analysis with g*Power (Kang, 2021). With our sample size *n* = 26, α = 0.05, power = 80%, we had the sensitivity to detect an effect size *f* = 0.189 for 2 × 2 × 2 ANOVA, *f* = 0.235 for 2 × 2 ANOVA, and *d* = 0.571 for a pairwise t-test comparison.

### Apparatus, stimuli, and procedure

Participant’s dominant hand and face were photographed within a controlled laboratory setting. The hand image, captured from a first-person perspective, underwent a grayscale transformation, cropping, and 180° rotation to create a third-person perspective copy. A face and a hand from the previous same-gender and hand-laterality participant, around the same age, were selected as other stimuli. Thus, target stimuli comprised self and other hands presented in first or third-person perspective, whereas prime stimuli were self and other faces (2 × 2 × 2 design). The experiment was programmed using MATLAB (2021b) and Psychtoolbox (Brainard, 1997) and performed on a BenQ Monitor (1.920 × 1.080 pixel resolution, 120 Hz, 24”) at a distance of 57 cm. Participants’ head position was stabilized by a chinrest with a custom built-in stereoscope able to guarantee a stable binocular vision after ad hoc adjustments.

**Fig. 1:**
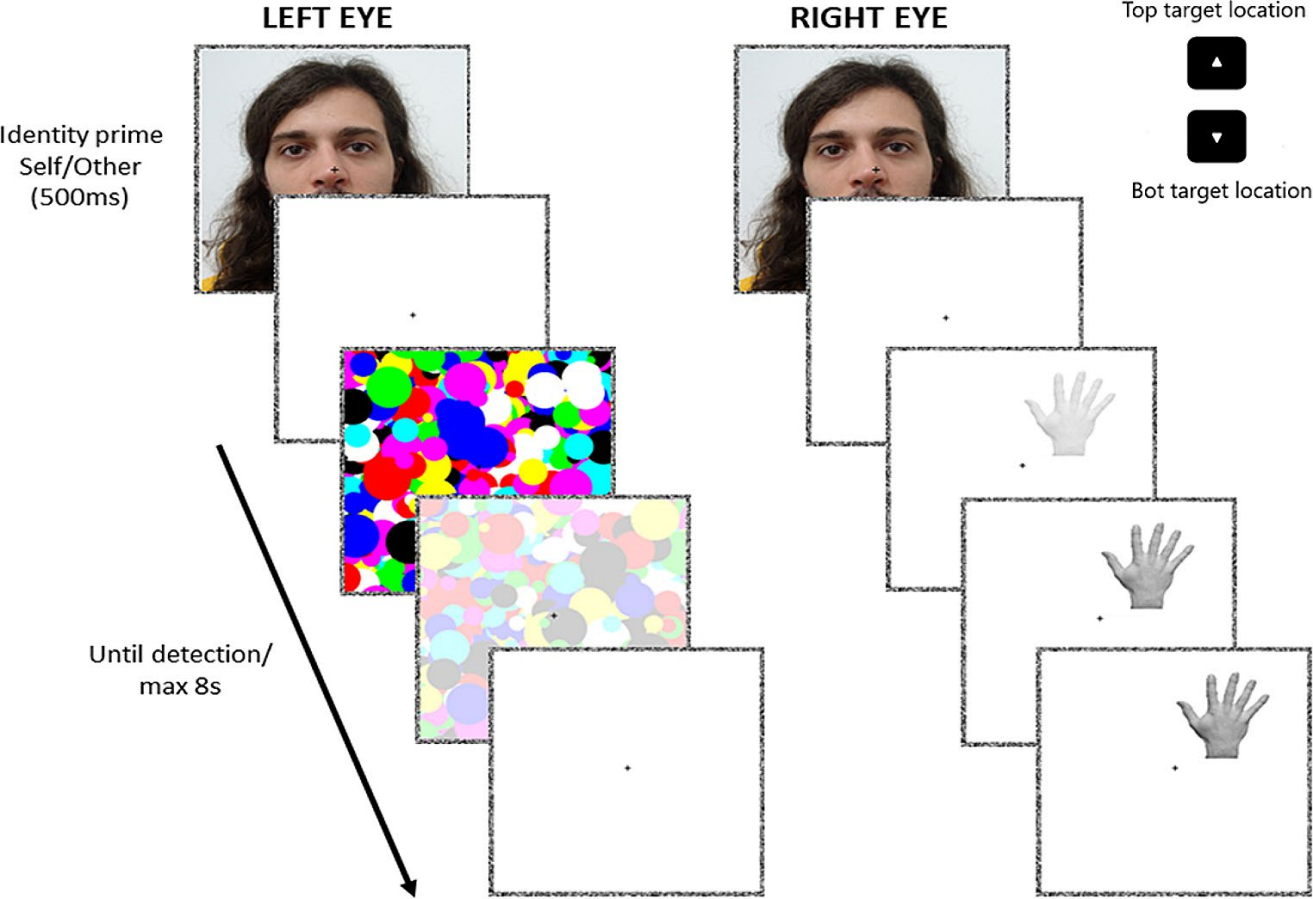
Schematic representation of the bCFS trial. Trials started with 500 ms of binocular prime presentation (self-other face). Then, the dynamic Mondrian pattern (10 Hz) is shown to one eye, being fully visible for 1 s and then linearly decreasing it to 0% decreased 7 s. At the same time, the target hand is shown to the other eye, being initially invisible but linearly increasing its visibility to 100% in 1 s. Each trial lasted for a maximum of 8.5 s or until response for target localization pressing the corresponding arrow as fast as possible. 1 s of ITI divided each trial

Before starting the experiment, participants underwent a stimuli familiarization and discrimination procedure. They were exposed to the target hand stimuli and the faces and were asked a) whether they could discriminate between the own and the other (changing the stimuli if not, but that was never the case), and b) to carefully observe other stimuli until they felt familiarized with (*intra-experimental* familiarity (Ramon & Gobbini, 2018). The experiment was structured as follows: within a black screen background, each trial started with a conscious exposition of the prime face (self/other; 11.5° x 11.5°) binocularly in the two fusion squares (11.7° x 11.7° at 5.8° from the center each, made of a noise-pixels border of.2° width and a white area, with a black fixation-cross in the center) for 500 ms. Following the prime, a target hand (3° x 4.2°) was presented in one fusion square (i.e., to one eye), and a high-contrast Mondrian-pattern mask (i.e., randomly arranged circles of distinct colors, and sizes between 0.3 and 1.2°, flashed at 10Hz, 11.5° x 11.5°) to the other. The target appeared by linearly decreasing its transparency from 100–0% within the first second of trial, presenting it at the top or the bottom of the fusion square (with a random horizontal jitter). Together, the transparency of the mask was linearly increased from 0–100% within the seven seconds after the first. Participants were instructed both verbally and in a written form (i.e., a sheet of paper, in Italian, which is available upon request to L.P.). They were instructed that (prime) faces were not directly related to the bCFS localization task, but they were nevertheless asked to keep in mind the face identity while localizing the suppressed target hand, as sometimes, at the end of a trial, they could have been asked to correctly select the prime that preceded the localization task (each then shown for 500 ms, divided by 650 ms; left arrow for the first, right arrow for the second). Prime-check trials consisted of the 12.5% of the total trials (48) and were included in the experiment to enhance the priming effect and to induce the participant to carefully pay attention to them. After the prime face presentation, participants were instructed that a target hand would have been presented, in a jittered horizontal position, at the top or bottom location of the fixation cross, that they could not consciously perceive immediately, but possibly after a variable time. They were asked to reply as fast and accurately as possible by localizing the position pressing the keyboard arrows (i.e., top-position: up arrow key, bottom-position: down arrow key) once it broke the mask’ suppression. It was also pinpointed to answer whether they had a strong feeling that something more than the mask was present in such location (i.e., preventing stimuli conscious perception). If they could not perceive the hand, no response was required. Importantly, their eyes had to be kept on the central fixation cross for the whole trial, it was forbidden to close one eye only, and also eye-blinking was, if possible, allowed after response. The trial ended with participant’s response or lasted for 8 s maximum, and 1 s of inter-trial interval delayed the next trial. Within each condition, stimuli were randomly administered 24 times to the right eye in a top position, 24 times to the right eye in a bottom position, 24 times to the left eye in a top position, and 24 times to the left eye in a bottom position for a total of 96 trials. Primes were also randomized across the trials, with half consisting in self-face primes and the other half other-face primes. Thus, the total experiment consisted of 384 trials, divided in 3 blocks of 128 trials, after each a small break was given (see Fig. 1 for time course of a trial and stimuli). The experiment started after eight familiarization trials with 4 random prime-check trials.

## Statistical analysis

No participants reported either instable binocular perception or a prime-check accuracy lower than 75% (i.e., no attention to primes), whereas one was excluded because of an accuracy lower than 90%, as a widespread practice in bCFS studies. The final sample consisted of 26 participants. Trials with a response time lower than 300 ms (3.9% of the trials) were excluded since they indicated that stimuli were not suppressed. Then, for each of the eight conditions, mean response times for corrected responses only were calculated, and then log-transformed because of a not normal data distribution (Shapiro-Wilk < 0.05). A repeated measure ANOVA with the factors Prime (self/other-face), Identity (self/other hand) and Perspective (first-/third-person) was run with JASP (JASP Team, 2016). Then, to further analyze our data, given the main effect of Perspective and the 3-way interaction, we run two separate repeated measures ANOVA for each perspective to evaluate how the factors Prime (self/other-face) and Identity (self/other hand) impacted on each perspective. For non-significant results, we analyzed to what extent the evidence supported the null hypothesis model (expressed in BF_01_) through Bayesian analysis (Cauchy distribution = 0.707).

## Results

**Fig. 2:**
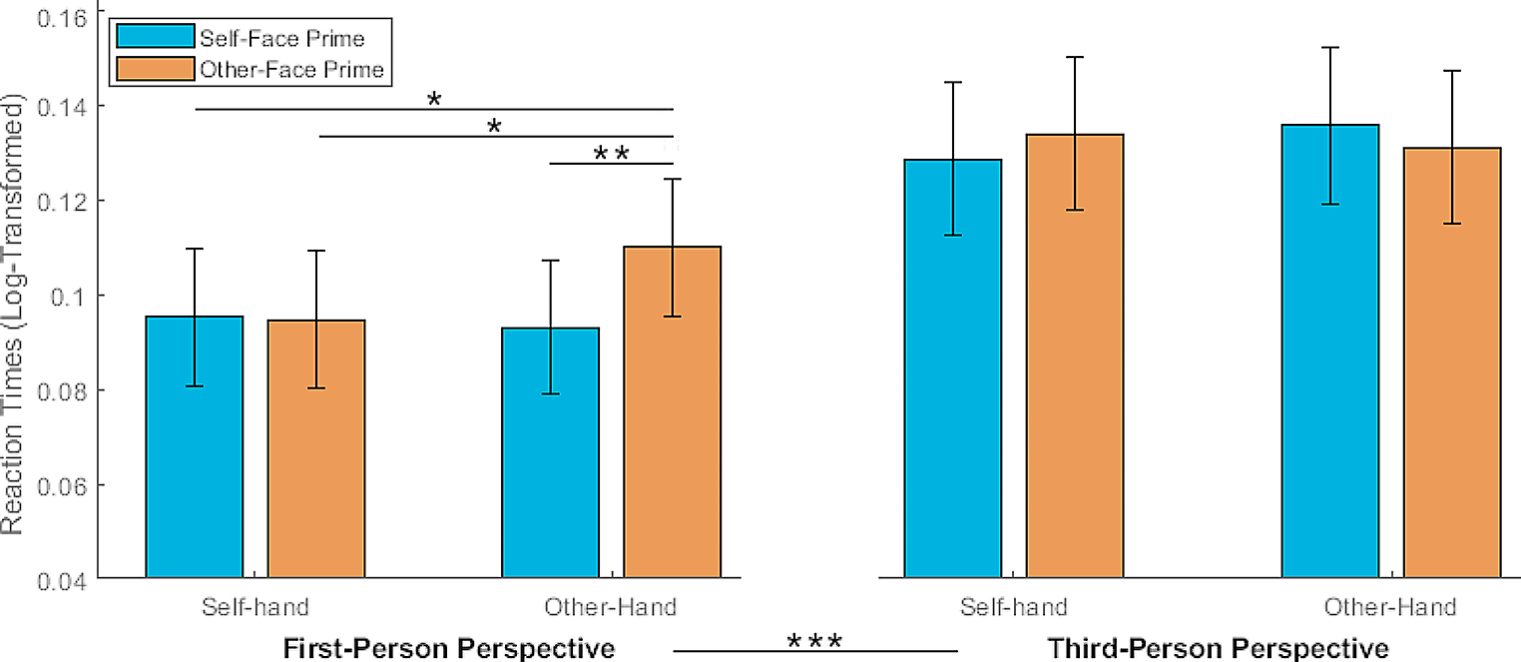
Results. On the left, mean log-transformed response time for First-Person perspective as a function of hand identity and prime (and SE), indicating the timing of target suppression. *p < .05, **p < .01, ***p *< .001*

Mean accuracy for the localization task was 0.96, while mean prime-check accuracy was 0.96. Mean response times for correct response before the log-transformation were 1.57 s (SE = ± 0.13). The 2 × 2 × 2 repeated measure ANOVA revealed a main effect of perspective (F_(1,25)_ = 22.06, *p* < .001, η_p_^2^ = 0.468), with significantly (*t*_(25)_ = -4.65, *p* < .001, Cohen’s d = 0.24) faster responses to first (mean_(log)_ = 0.09, SE = ± 0.03), than third (mean_(log)_ = 0.13, SE = ± 0.03) person perspective (see Fig. 2). Moreover, a triple interaction between Prime x Identity x Perspective was found (F_(1,25)_ = 5.71, *p* = .025, η_p_^2^ = 0.186). As regards the two separate repeated measures ANOVA, we found within the First-Person perspective, a significant interaction (F_(1,25)_ = 6.64, *p* = .016, η_p_^2^ = 0.210) with slower response to other-hands primed with other faces as compared to all the other conditions, namely other-hands primed with the self-face (*t*_(25)_ = 3.01, *p* = .006, Cohen’s *d* = 0.59), self-hands primed with other face (*t*_(25)_ = 2.13, *p* < .043, Cohen’s *d* = 0.49), and self-hand primed with self-face (*t*_(25)_ = 2.11, *p* < .045, Cohen’s *d* = 0.41). None of the main factors (i.e., Prime and Identity) were significant (*p* > .05, BF_01_ = 1.99, BF_01_ = 3.11, respectively). The same analysis on the Third-Person perspective condition yielded no significant effects (*p* > .05; BF_01_ = 3.36 for the factor Prime, BF_01_ = 3.40 for the factor Identity, BF_01_ = 24.01 for the interaction). See Fig. 2.

Summarizing, these results show that first-, with respect to the third-, person perspective broke the suppression faster. Moreover, in first-person perspective other-hands stimuli preceded by other-face prime broke the suppression slower with respect to other-hands stimuli preceded by self-face prime, self-hands stimuli preceded by other-face prime, self-hands stimuli preceded by self- face prime.

## Discussion

By means of the breaking-Continuous Flash Suppression paradigm (bCFS), here we investigated whether and to which extent conscious identity primes acted upon perspective and identity cues affecting the access to awareness timing of hand stimuli. We found that the first-, as compared to the third- person perspective, speeded up the response. Crucially, we also reported that, within the first-person perspective, other-hands stimuli preceded by other-face priming slowed down the responses with respect to the other conditions.

The main effect of perspective without a main effect of identity, replicates the results of a previous study (Ciorli & Pia, 2023), confirming that the perspective from which the own body is typically perceived is prioritized before awareness. As extensively discussed in that paper, this represents (behaviorally) a mechanism to support action monitoring via the generation of a stable visuospatial egocentric representation. The lack of an identity effect is simply a residual of the optimization of the process so that such direct body coding suppresses irrelevant information, which, on the other hand, could be useful at later stages. This pattern is not trivial but, rather, consistent with other literature related to ownership attribution, based on bodily illusion. Indeed, these experimental manipulations show that external objects as, for instance, fake hands (Botvinick & Cohen, 1998; Costantini & Haggard, 2007; Kalckert & Ehrsson, 2014; Pyasik et al., 2019) or virtual bodies (Maselli & Slater, 2013; Petkova & Ehrsson, 2008; Pyasik et al., 2022; Romano et al., 2014) are misattributed to the self if stimuli are presented in a first-person perspective regardless its visual appearance (i.e., identity). However, in the present study we did report an unconscious effect of stimulus identity only when a conscious identity-related prime (i.e., faces) was provided. Specifically, we reported that in first-person perspective, other-hands stimuli preceded by other-face priming slowed down the responses with respect to other-hands stimuli preceded by self-face priming, and to self-hands stimuli preceded by self- or other- face priming. How can we put together these findings? In the remaining part of the paper, we will attempt to provide an explanation.

A possible useful framework is the predictive coding account of self-recognition (Apps & Tsakiris, 2014; Tsakiris, 2017). It is stated that the own body emerges as a probabilistic representation (i.e., the most likely to be ‘me’ object) from the interplay between body priors and actual incoming body-related sensory signals. In details, priors trigger predictions about the sensory consequences subsequently evoked on the body by incoming stimuli. When predicted and actual consequences match each other’s the process ends, whereas when there is a mismatch between the two, priors must be updated. Importantly, predictions do not have the same relevance since ambiguous signals require greater efforts and inferences to update priors. We argue that the main effect of perspective, and the lack of the identity effect, reflects the fact that predictions rooted on perspective, being highly unambiguous (i.e., it is highly likely that the self-hand appears in the first-person perspective), overwhelm those created from identity (i.e., visual features can vary along a wide range of states). This mechanism is highly adaptive being able to overcome the frequent changes of perceptual features of the own hand (e.g., wearing gloves), whereas the point of view is fixed across the experience. In other words, perspective cues are sufficient in several daily-life situations. As for the conscious priming, it is worth of noticing that a prime provides per se additional cues that can potentially act upon the ongoing unconscious processes in a top-down fashion. Not surprisingly, here priming affected the unconscious response only within the first-person perspective, namely the level of the only variable displaying an unconscious advantage. However, self-face priming provided unambiguous information (i.e., self-face) so that these cues are suppressed because redundant, and perspective remain sufficient to unconsciously attribute the hand to the self (even if it belonged to another person). The prime exerted an effect only in the most ‘critical’ condition, namely with other-hands stimuli, and not with self-hand stimuli. In other words, when both the conscious prime and stimulus belonged to another person were administered, the first-person advantage was lost because of the presence of the highest degree of ambiguity. In short, priming resulted effective only when the probability of that stimulus to be unequivocally coded as self-related was the lowest. This, in turn, slowed down the conscious access possibly because of a prediction update process. Interestingly, this is in line with the idea that the content of visual awareness emerges once prediction errors are minimized through the prediction and sensory evidence verification cycle, and with the fact that, under conditions of stimulus ambiguity, not only prior beliefs act upon stimulus prioritization, but also prioritization can be extended to other-related stimuli (Falben et al., 2020).

Summarizing, our results suggest that self-attribution of bodily stimuli before visual awareness relies on spatial perspective except for very ambiguous circumstances that necessitate higher order resources. These findings extend the link between ownership attribution, self-relevance prioritization effects, and the predictive processing hypothesis, highlighting the importance of top-down feedback projections for the content of conscious perception (Hohwy & Seth, 2020; Lamme & Roelfsema, 2000). Additionally, our findings suggest that the visual system in general gives precedence to corporeal stimuli belonging to the self as compared to another person. Interestingly, this is consistent with the Self-Attention Network (Sui & Humphreys, 2015) showing that self-related information enjoy enhanced perceptual processing as a consequence of bottom-up attentional capture or higher order mechanisms such as, for instance, decision making or memory, here capitalized by spatial perspective but not identity.

Before concluding, some limitations of the study should be emphasized. First, we tried to control for the well-known role of low-level features on unconscious processing by creating stimuli under rigorous experimental constraints. Most importantly, stimuli were the same in the two perspectives and self-hand stimuli were used as other-hand stimuli for the subsequent participant (i.e., we alternated the same stimuli, keeping the same low-level features and only changing their high-level meaning in the eye of the observers). One might still argue that the different (i.e., mirror) shade due to a 180° rotation could have had a role. Despite we cannot exclude such option, it fails to explain the interaction we reported here. Second, self-stimuli constitute the most familiar perceptual objects of our existence, thus making hard per se to control for visual familiarity that might influence the results. Here we adopted experimental rather than personal familiarity, thus whereas we can conclude that this effect is not driven by the mere visual familiarity, how personal familiarity could act on the investigated mechanism is worth investigating. Moreover, the interaction we report is certainly driven also by other-related features (i.e., other face and hand). Third, it is worth noticing that the priming task we adopted here differs from classical approaches being primes consciously perceived and objects of a memory task in prime-check trials, unlike usual priming tasks in which primes are even unaware or ignored. We adopted such solution to investigate conscious priming effects on visual conscious access, as in Stein and colleagues’ work (Stein et al., 2023). As the conscious access indexed by the bCFS paradigm is particularly sensitive to the working memory content (Pan et al., 2014), we’ve added such task to maximize top-down prime effects on visual processing. It cannot be excluded that such approach was crucial for our findings, or whether the same results could have been obtained with more classical approaches. A fourth limit regards the absence of an object condition as a prime or, more importantly, as a target. Consequently, our study focusing on bodily attribution cannot generalize its findings to the general category of self-associated stimuli, an interesting topic worth of future investigations. Similarly, being bCFS rooted on a conscious measure of subjective visibility, it cannot exclude response biases (Stein, 2019) and, consequently, more stringent techniques relying on objective awareness measures (Stein & Peelen, 2021), could be employed to expand our findings.

## Data Availability

The data that support the findings of this study are available on request from the corresponding author L.P.
